# Synergistic In Vitro Antimicrobial Activity of Triton X-100 and Metformin against *Enterococcus faecalis* in Normal and High-Glucose Conditions

**DOI:** 10.3390/microorganisms10010124

**Published:** 2022-01-07

**Authors:** Xinling He, Siqi Jin, Wei Fan, Bing Fan

**Affiliations:** The State Key Laboratory Breeding Base of Basic Science of Stomatology (Hubei-MOST) and Key Laboratory of Oral Biomedicine Ministry of Education, School and Hospital of Stomatology, Wuhan University, Wuhan 430079, China; 2014302220020@whu.edu.cn (X.H.); 2018303042023@whu.edu.cn (S.J.)

**Keywords:** antimicrobial activity, dentinal tubules, *Enterococcus faecalis*, high-glucose condition, metformin, Triton X-100

## Abstract

The prevention and treatment of oral diseases is more difficult in diabetic patients with poorly controlled blood glucose levels. This study aims to explore an effective, low-cytotoxicity medication for root canal treatment in diabetic patients. The antibacterial effect of the combination of Triton X-100 (TX-100) and metformin (Met) on *Enterococcus faecalis* (*E. faecalis*) was evaluated by determining the minimum inhibitory concentration (MIC), minimum bactericidal concentration required to kill 99% bacteria (MBC_99_) and by conducting dynamic time-killing assays. While the antibiofilm activity was measured by crystal violet (CV) assay, field emission scanning electron microscope (FE-SEM), confocal laser scanning microscope (CLSM) and colony-forming unit (CFU) counting assays. The expression of relative genes was evaluated by real-time quantitative polymerase chain reaction (RT-qPCR), and the cytotoxicity of the new combination on MC3T3-E1 cell was also tested. Results showed that the antibacterial and antibiofilm activities of Met could be significantly enhanced by very low concentrations of TX-100 in both normal and high-glucose conditions, with a much lower cytotoxicity than 2% chlorhexidine (CHX). Thus, the TX-100 + Met combination may be developed as a promising and effective root canal disinfectant for patients with diabetes.

## 1. Introduction

*Enterococcus faecalis* (*E. faecalis*), a Gram-positive facultative anaerobe, has been highly detected in refractory root canal infection and reinfection of human teeth [1,2,3,4]. As such, it has often been used to evaluate the antibacterial efficiency of various intracanal medications [5,6]. In diabetic patients, high blood glucose levels provide rich nutrients for bacteria to survive and proliferate in infected areas. Therefore, uncontrolled diabetes is associated with low oral health-related quality of life [7], with patients more likely to develop oral diseases [8,9,10,11]. The prevention and treatment of oral diseases in diabetic patients with poorly controlled blood glucose levels would be more difficult than in those with well-controlled blood glucose levels [12,13].

The curvatures and complex internal anatomical variations of the root canal system make it difficult for the chemomechanical preparation of root canal treatment to fully achieve the treatment goals [14]. In addition, it may be difficult for the disinfectant to reach the target lesion at a fully effective concentration, due to the narrow anatomical structure of the root. However, subinhibitory concentrations of antibiotics in the variant anatomy of the root canal may in turn induce increased biofilm formation ability and decreased antibiotic susceptibility of *E. faecalis* [15], and finally lead to the failure of endodontic therapy. Moreover, antibiotic resistance of many pathogenic bacteria is becoming a serious threat to clinical treatment and public health [16,17]. Therefore, non-antibiotic bactericidal agents have been the focus of research in recent decades. 

Metformin (Met) is commonly used to treat type 2 diabetes mellitus [18,19,20]. It is generally believed that the metabolic regulatory effect of Met is related to the activation of adenosine monophosphate-activated protein kinase. By inhibiting complex I of the mitochondrial respiratory chain, Met further inhibits the production of adenosine triphosphate, thereby reducing gluconeogenesis [21,22]. As a clinical medicament, Met shows pleiotropic effects, such as direct inhibition of inflammation [22], protection of the cardiovascular system [23], and inhibition of tumor growth, in addition to its traditional effects on glucose metabolism [24,25]. As an antibacterial agent, Met has been shown to be effective against a wide range of microbes, including oral pathogens [21,26]. Khajuria et al. [27] demonstrated its inhibitory activity against *Porphyromonas gingivalis* and hence its effectiveness in the treatment of diabetes-related periodontitis. However, to date, no studies have investigated the inhibitory effect of Met on *E. faecalis* in root canal infections, especially in patients with diabetes. 

Triton X-100 (TX-100), a non-ionic surfactant, is commonly used to increase the cell membrane permeability to antibodies but itself has only weak antibacterial activity [28,29]. Some studies have used TX-100 to modify nanomaterials so as to enhance their antibacterial effects [28,30]. Some researchers have combined TX-100 with other materials to obtain synergistic antimicrobial effects [31,32,33]. Komatsuzawa et al. [34] reported that TX-100 could alter the drug resistance of *Staphylococcus aureus*.

Based on the above information, the main objective of this study was to combine the non-ionic surfactant TX-100 and Met and investigate the synergistic antimicrobial effect of the new combination against *E. faecalis* in both normal and high-glucose conditions.

## 2. Materials and Methods

### 2.1. Antibacterial Activities of TX-100, Met, and TX-100 + Met against Planktonic E. faecalis

#### 2.1.1. Minimum Inhibitory Concentration (MIC) and Minimum Bactericidal Concentration Required to Kill 99% Bacteria (MBC_99_)

The MIC and MBC_99_ of TX-100 and Met were determined using a serial microdilution assay. Briefly, a series of two-fold dilutions of TX-100 (neoFroxx GmbH Co., Ltd., Einhausen, Germany) (ranging from 10.24% to 0.02%) and a series of solutions of Metformin hydrochloride (Aladdin Industrial Corporation Co., Ltd., Shanghai, China) with a concentration interval of 40 mg/mL (ranging from 120 to 400 mg/mL) were prepared in sterile double-distilled water. For the culture of *E. faecalis*, normal brain heart infusion (BHI) medium was used to simulate normal conditions, while BHI supplemented with glucose (final concentration is 25.5 mM) was used to simulate high-glucose conditions. Subsequently, 100 μL suspension [2 × 10^5^ colony-forming units (CFU)/mL] of *E. faecalis* (ATCC 29212, ATCC, Manassas, VA, USA) in BHI medium (normal and high-glucose) and 100 μL of drug solution as described above were mixed in a 96-well plate, and then incubated in a humidified atmosphere of 5% CO_2_ and 1% O_2_ at 37 °C (same for all the following *E. faecalis* experiments) for 24 h. Untreated bacteria and BHI medium were used as blank and background controls, respectively. The absorbance at 600 nm was measured using a microplate reader (Power Wave XS2, BioTek Instruments, Winooski, VT, USA). The MIC was defined as the lowest concentration with no turbidity compared to the background control. The MBC_99_ was determined by inoculating the suspensions at concentrations equal to or higher than the MIC on a BHI agar plate. The lowest concentrations at which less than 1% of the initially inoculated colonies of the blank control were visible after incubation were regarded as MBC_99_. This test was repeated six times. 

#### 2.1.2. Synergistic Antibacterial Activities of TX-100 and Met against *E. faecalis*

The synergistic MIC and MBC_99_ of TX-100 and Met were determined using the method described above. In addition, 0.02% and 0.04% concentrations of TX-100 were selected as the final synergistic concentrations, and a series of Met solutions with final concentrations ranging from 10 to 90 mg/mL was prepared. This test was repeated six times. 

#### 2.1.3. Dynamic Time-Killing Assays

The bacterial dynamic time-killing assay was performed using the following method. *Enterococcus faecalis* in the exponential phase was diluted in BHI to obtain a concentration of 2 × 10^6^ CFU/mL. For the single-drug test, 0.04% TX-100 and 100 mg/mL Met were used. For the combination drug test, TX-100 (final concentrations were 0.02% and 0.04%) and Met (final concentrations were 40, 60, 80, and 100 mg/mL) were mixed in advance. Thereafter, 500 μL aliquots of the solutions containing single or combination drugs at different concentrations were mixed with the same volume of the diluted bacterial suspension, followed by incubation at 37 °C. Chlorhexidine (CHX, 2%) (Adamas, Basel, Switzerland) was used as a positive control, and non-treated groups were used as blank controls. At 3, 6, 12, 24, 36, and 48 h, the samples were agitated using a vortex mixer (Zhongxi Yuanda Technology Co., Ltd. Beijing, China), and 10 μL of the mixture was taken out and inoculated onto a BHI agar plate. After incubation at 37 °C for 24 h, the bacterial colonies of different groups were counted. The assays were performed six times. 

### 2.2. Antibiofilm Activities of TX-100, Met, and TX-100 + Met against E. faecalis Biofilm

#### 2.2.1. Inhibition of Bacterial Attachment

The effects of TX-100, Met, and their combinations on *E. faecalis* biofilm were determined using the following method. First, a series of final concentrations of Met solutions (0.5×, 1×, 2×, 3×, and 4× MIC) were prepared with or without TX-100 (0.02% and 0.04%). *Enterococcus faecalis* treated with BHI medium was used as a blank control. Subsequently, 100 μL of the test solution and 100 μL of *E. faecalis* suspension (2×10^8^ CFU/mL) in BHI medium (normal and high-glucose) were mixed in a 96-well plate and incubated at 37 °C for 24 h. The crystal violet (CV) assay was used to measure the biomass and metabolic activity of *E. faecalis* biofilms. Briefly, after incubation, the supernatant was discarded gently, and the biofilms were washed with phosphate-buffered saline (PBS) (pH 7.2) to remove unattached bacteria. After fixing with 200 μL of anhydrous methanol for 15 min, the biofilms were stained with 200 μL of 0.1% CV for 15 min in the dark. The unbound CV was washed with PBS, and the biofilm-bound CV was dissolved in an additional 200 μL of anhydrous methanol. A microplate reader was used to measure the absorbance of the bound CV at 560 nm. The lowest concentration at which biofilm formation was inhibited by at least 50% compared to that of the blank control was determined as the minimum biofilm inhibition concentration (MBIC_50_). The experiment was repeated six times. 

#### 2.2.2. Biofilm Disruption Assay

This assay was similar to that described above. Briefly, 200 μL of *E. faecalis* suspension (2×10^8^ CFU/mL) in BHI medium (normal and high-glucose) was seeded into a 96-well plate and incubated at 37 °C for 72 h for biofilm formation. Subsequently, the supernatant was replaced with the test solutions prepared in BHI medium, followed by another 24 h of incubation for biofilm disruption. The absorbance of the biofilm-bound CV measured at 560 nm was used to evaluate the effect of the test solutions on the biofilm. The lowest concentration at which the formed biofilm was eradicated by at least 50% compared to the blank control was determined as the minimum biofilm reduction concentration (MBRC_50_). The assay was repeated six times. 

### 2.3. Antimicrobial Effect against E. faecalis on Dentin

Dentin slices (4 mm wide × 4 mm long × 1 mm thick) were prepared from human extracted wisdom teeth. All dentin slices were ultrasonically rinsed for 4 min in double-distilled water, 5.25% sodium hypochlorite (NaClO), and 17% ethylenediaminetetraacetic acid (EDTA), and then again rinsed in double-distilled water for 1 min. The dentin slices were autoclaved at 121 °C for 20 min in double-distilled water and then incubated for 24 h in BHI at 37 °C to ensure aseptic conditions. Sterilized dentin slices were soaked in 1 mL of *E. faecalis* suspension (1×10^8^ CFU/mL) in BHI medium (normal and high-glucose) and then incubated at 37 °C for 28 days for allowing biofilm formation on dentin. To ensure bacterial viability, fresh BHI medium was replaced every 72 h. 

Subsequently, the incubated dentin slices were washed with sterile PBS to remove unattached bacteria, immersed in the test gel, and then incubated at 37 °C. Briefly, 2 mL of the test solutions of different concentrations, prepared in PBS, were mixed with 0.15 g of carboxymethyl cellulose sodium (Aladdin Biochemical Technology Co., Ltd. Shanghai, China) to form gels. Based on the aforementioned experimental results, three concentrations (0.04% TX-100 + 60 mg/mL Met, 0.04% TX-100 + 80 mg/mL Met, and 0.04% TX-100 + 100 mg/mL Met) with the best antimicrobial effect were used in this antimicrobial test on dentin. A gel with 2% CHX was used as a positive control and a gel with PBS was used as a blank control. The gel treatment was performed for 7 days to simulate clinical root canal medication. 

#### 2.3.1. *E. faecalis* on the Dentin Surface

In order to evaluate the corrective effect of the gel treatment on the *E. faecalis* on the dentin surface, two dentin slices from each group were randomly selected for observation by field emission scanning electron microscope (FE-SEM; Ultraplus; Zeiss, Oberkochen, Germany), and six slices were washed with PBS to remove remnant gels before being transferred into 10 mL of fresh BHI medium (normal and high-glucose) at 37 °C. At 2, 4, 6, 8, 10, 12, and 24 h after incubation, 1 mL of suspension was taken out for measuring absorbance at 600 nm with a spectrophotometer (UV-2401PC, Shimadzu Corp., Kyoto, Japan). 

#### 2.3.2. *E. faecalis* in the Dentinal Tubules

Confocal laser scanning microscope (CLSM) and CFU counting method were used to evaluate the antibacterial effect of the gel treatment on *E. faecalis* that invaded into the dentinal tubules. Three dentin slices selected randomly from both normal and high-glucose BHI were split to expose the longitudinal section of the dentinal tubules, and then was stained with LIVE/DEAD^®^ BacLight™ Bacterial Viability Kit (Invitrogen, Waltham, MA, USA). After that, the penetration depth of *E. faecalis* in the dentinal tubules was examined by an inverted Leica TCS SP8 confocal microscope (Leica Microsystems, Mannheim, Germany). Fluorescence images were analyzed with Leica confocal software LAS X (Leica Microsystems). Ten randomly selected images from each specimen were examined and the largest distance from the dentin surface to the visible bacteria was determined using the measurement function. Then, 30 quantitative data per group were collected for statistical analysis. The dentin powder samples used for CFU counting were obtained following the protocol described in previous studies [35,36,37] with some modifications. Three dentin slices from each group were washed with PBS and gently curetted to remove residual gel and bacteria on the surface. Then, a sterile round bur (ISO size: 012) mounted in a handpiece was used to collect dentin powder samples at low speed. Sterile forceps were used to maintain the specimens in place during sampling. The *E. faecalis* from the dentinal tubules were collected by centrifugation and resuspension in 1 mL BHI. The dentin powder samples were vigorously mixed by vortex for 20 s before and after incubation for 1 h at 37 °C, and then transferred to a BHI agar plate for CFU counting. 

### 2.4. In Vitro Gene Expression Assay

*Enterococcus faecalis* (10^8^ CFU/mL) treated with 0.02% TX-100, 20 mg/mL Met, or 0.02% TX-100 + 20 mg/mL Met (MIC) were co-incubated at 37 °C for 24 h. Untreated *E. faecalis* was used as the blank control. RNA extraction was performed as described previously. Four milliliters of *E. faecalis* were harvested by centrifugation (3000× *g*, 6 min, 4 °C) and then resuspended in 0.5 mL of lysozyme (20 mg/mL) at 37 °C for 15 min to liberate the cell wall. Lysozyme was removed by centrifugation (3000× *g*, 6 min, 4 °C) and replaced with 1 mL of TRIzol Reagent (Invitrogen, Carlsbad, CA, USA), followed by incubation for 10 min. The resulting solution was mixed with 200 μL of chloroform, vortexed for 15 s, and incubated for 5 min. After centrifugation (12,000× *g*, 15 min, 4 °C), the RNA in the aqueous phase was transferred to a new tube and mixed with an equal volume of isopropanol (4 °C), followed by incubation on ice for 10 min. RNA was collected by centrifugation (12,000× g, 10 min, 4 °C), washed twice with 1 mL of 75% ethanol (4 °C), and air-dried before being resuspended in 20 μL of diethylpyrocarbonate (DEPC)-treated water. A UV spectrophotometer (NanoDrop, 2000; NanoDrop Technologies, Wilmington, DE, USA) was used to determine the RNA concentration and purity. 

Reverse transcription was performed as described previously. One thousand nanograms of RNA from each sample was used as the template, and reverse transcription polymerase chain reaction (PCR) was performed using a Mastercycler (Eppendorf, Hamburg, Germany) with the HiScript^®^ II Q RT SuperMix for qPCR Kit (Vazyme Biotech Co., Ltd., Nanjing, China).

Real-time quantitative PCR (RT-qPCR) was performed using the ABI QuantStudio 6 Flex (Applied Biosystems, Waltham, MA, USA) with the ChamQ Universal SYBR qPCR Master Mix Kit (Vazyme Biotech Co., Ltd., Nanjing, China). The PCR program settings were as follows: hold stage (95 °C for 3 min), PCR stage (40 cycles at 95 °C for 10 s and 60 °C for 30 s), and melt curve stage (95 °C for 15 s, 60 °C for 1 min, and then 95 °C for 15 s). The primers used in this study are listed in Table 1. To standardize the expression level of the target gene, the 16S rRNA gene was selected as the housekeeping gene. TX-100 (0.02%) and Met (20 mg/mL) were used to treat single-drug groups, and 0.02% TX-100+20 mg/mL Met was used to treat the synergistic drug groups. Untreated *E. faecalis* was used as a blank control and as a baseline to compare gene expression. The raw threshold cycle (Ct) values were calculated for relative expression using the formula 2^−ΔΔCt^. Three independent replicate experiments were performed. 

### 2.5. Cytotoxicity Test

To assess biocompatibility, the cell counting kit-8 (CCK-8) method was performed on MC3T3-E1 cells (ATCC). Different concentrations of test solutions, as described in *Dynamic time-killing assays*, were prepared and then sterilized using a 0.22-μm-pore-size filter unit (Merck Millipore Ltd., Darmstadt, Germany). For cell culture, 1×10^4^ cells suspended in 100 μL α-Modified Eagle’s Medium (MEM; Thermo Scientific, Waltham, MA, USA) with 10% fetal bovine serum (FBS; Thermo Scientific) and 1% penicillin/streptomycin (Thermo Scientific) were inoculated into each well of a 96-well plate and incubated at 37 °C for 24 h. Subsequently, the supernatant was replaced with 200 μL of fresh normal and high-glucose Dulbecco’s Modified Eagle’s Medium (DMEM; HyClone, Logan, UT, USA), and 10 μL of test solutions were added for another 24-h incubation at 37 °C. Cells exposed to 2% CHX were used as the positive control, non-treated cells were used as the blank control, and wells containing medium only were used as the background control. Each group included six replicate wells. The cells were washed with PBS and then incubated with 100 μL of α-MEM and 10 μL of CCK-8 solution (CCK-8, Dojindo Laboratories, Kumamoto, Japan) at 37 °C for 1.5 h in the dark. The absorbance was measured at 450 nm using a microplate reader. 

### 2.6. Statistical Analysis

GraphPad Prism 8 (San Diego, CA, USA) for Microsoft Windows was used for the statistical analysis. Data in normal distribution were analyzed using one-way analysis of variance with a Holm–Sidak’s multiple comparisons test or Student’s t-test. Data violating normality were analyzed using the non-parametric Kruskal–Wallis analysis with a Dunn’s multiple comparisons test. The mean±standard error (SEM) was used for normally distributed data, while the median±quartile deviation (i.e., P25–P75) was used for data violating normality. Statistical significance was set at *p* < 0.05. 

## 3. Results

### 3.1. Antibacterial Activities of TX-100, Met, and TX-100 + Met against Planktonic E. faecalis

The MIC and MBC_99_ values of the different combinations of TX-100 and Met are shown in Table 2. Met exhibited antibacterial activity against *E. faecalis*, with the same MIC value in normal and high-glucose BHI medium. The higher MBC_99_ value of Met against *E. faecalis* in high-glucose BHI than that in normal BHI indicated that *E. faecalis* in high-glucose condition may be more resistant to Met. In the presence of 0.02% and 0.04% TX-100, the MIC and MBC_99_ values of Met were five times lower, in both normal and high-glucose conditions, demonstrating the synergistically enhanced antibacterial effect of TX-100 and Met. In addition, TX-100 showed limited antibacterial ability when used alone, as no MIC and MBC_99_ values could be determined. 

**Figure 1:**
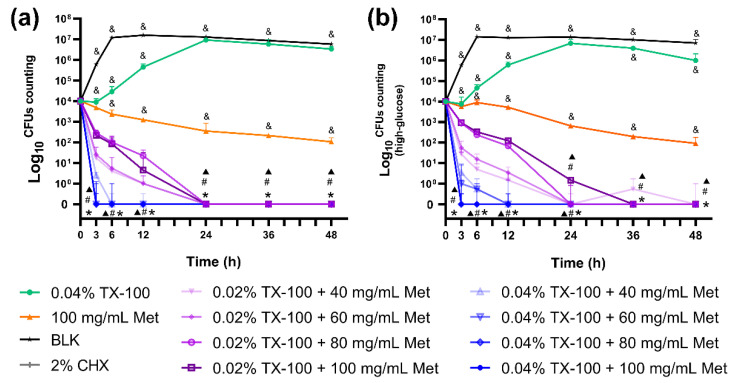
Dynamic time-killing curves. (**a**) in normal BHI; (**b**) in high-glucose BHI. (*: significant difference compared with the blank control group; ▲: significant difference compared with the 0.04% TX-100 group; #: significant difference compared with the 100 mg/mL Met group; &: significant difference compared with the 2% CHX group; *p* < 0.05).

In the dynamic time-killing assays, similar to the blank control, the group treated with TX-100 resulted in an exponential increase in bacterial growth because of its limited antibacterial activity. The number of colonies in the Met group remained almost constant because of its limited antibacterial activity. However, the bacteria were almost eliminated after 24 h by the co-use of 0.02% TX-100 and Met, showing a statistical difference compared to the single-drug and blank control groups (*p* < 0.05; Figure 1). Moreover, the bacteria were almost eliminated in the first 3 h due to the rapid bactericidal effect of the combination of 0.04% TX-100 and Met, showing no statistical difference when compared with the 2% CHX group (Figure 1).

### 3.2. Antibiofilm Activities of TX-100, Met, and TX-100 + Met against E. faecalis Biofilm

Compared to the single-drug treatment groups, the MBIC_50_ values of the synergistic groups were reduced by up to 10 times in normal BHI, and MBRC_50_ values were reduced by five times in both normal and high-glucose conditions (Table 3). In addition, *E. faecalis* biofilms seemed more robust in high-glucose BHI because of the higher MBRC_50_ values of TX-100 and Met in high-glucose condition. 

### 3.3. Antimicrobial Effect against E. faecalis on Dentin

**Figure 2:**
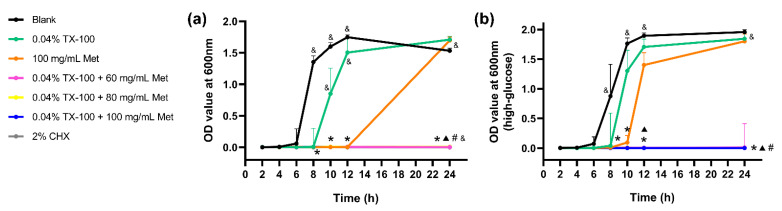
Growth curves of *E. faecalis* on dentin slices. (**a**) in normal BHI; (**b**) in high-glucose BHI. (*: significant difference compared with the blank control group; ▲: significant difference compared with the 0.04% TX-100 group; #: significant difference compared with the 100 mg/mL Met group; &: significant difference compared with the 2% CHX group; *p* < 0.05).

Similar to the blank control group, the *E. faecalis* bacteria on the dentin slices treated with single-drug showed an exponential growth during the 24-h incubation, exhibiting a significantly higher optical density (OD) value than the synergistic and positive control group (*p* < 0.05; Figure 2). The OD values of the synergistic groups treated with 0.04% TX-100+80 mg/mL Met, 0.04% TX-100+100 mg/mL Met, or 2% CHX showed no increase during 24 h of incubation in BHI (Figure 2), indicating that no live bacteria survived the 7-day treatment. Although the groups treated with 0.04% TX-100+60 mg/mL Met showed a slight increase in OD values, all synergistic groups in high-glucose BHI showed no statistical difference when compared with the 2% CHX group (Figure 2). 

**Figure 3:**
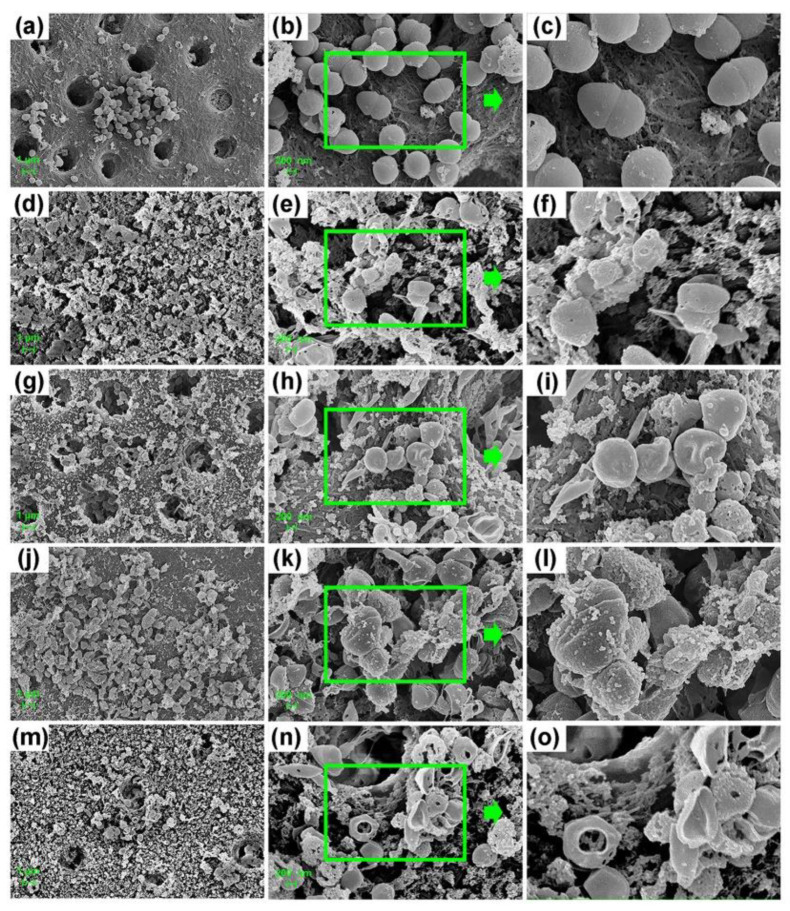
FE-SEM images of dentin biofilm in normal BHI. (**a**–**c**) Biofilm treated with PBS gel (blank control); (**d**–**f**) Biofilm treated with 0.04% TX-100 gel; (**g**–**i**) Biofilm treated with 100 mg/mL Met gel; (**j**–**l**) Biofilm treated with 2% CHX gel; (**m**–**o**) Biofilm treated with synergistic gel (0.04% TX-100 + 100 mg/mL Met) (magnifications are 5000×; 20,000×; zoom in).

**Figure 4:**
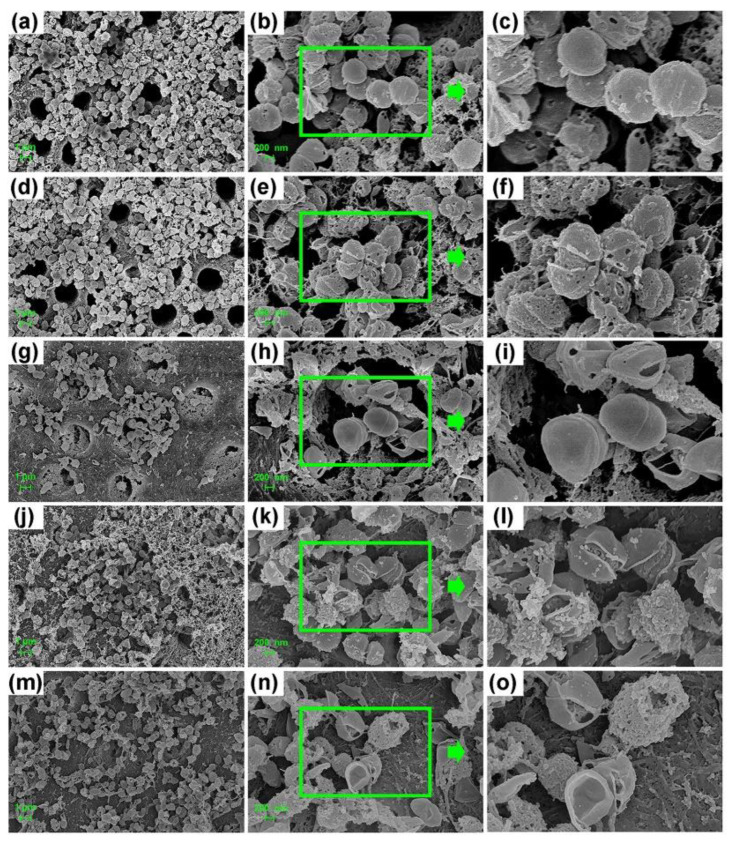
FE-SEM images of dentin biofilm in high-glucose BHI. (**a**–**c**) Biofilm treated with PBS gel (blank control); (**d**–**f**) Biofilm treated with 0.04% TX-100 gel; (**g**–**i**) Biofilm treated with 100 mg/mL Met gel; (**j**–**l**) Biofilm treated with 2% CHX gel; (**m**–**o**) Biofilm treated with synergistic gel (0.04% TX-100 + 100 mg/mL Met) (magnifications are 5000×; 20,000×; zoom in).

The FE-SEM images confirmed the differences in the morphology of bacteria on the dentin surface among different groups (Figure 3 and Figure 4). Extremely small perforations could be observed on the surface of *E. faecalis* in the 0.04% TX-100 group (Figure 3d–f and Figure 4d–f), while no significant change was observed in the 100 mg/mL Met group (Figure 3g–i and Figure 4g–i). However, the bacteria in the synergistic groups showed abnormal morphology as the cell membrane was ruptured or even dissolved with the cytoskeleton exposed (Figure 3m–o and Figure 4m–o). Although the morphology of *E. faecalis* in the 2% CHX group did not change significantly (Figure 3j–l and Figure 4j–l), the experiments showed that *E. faecalis* was not alive (Figure 2).

**Figure 5:**
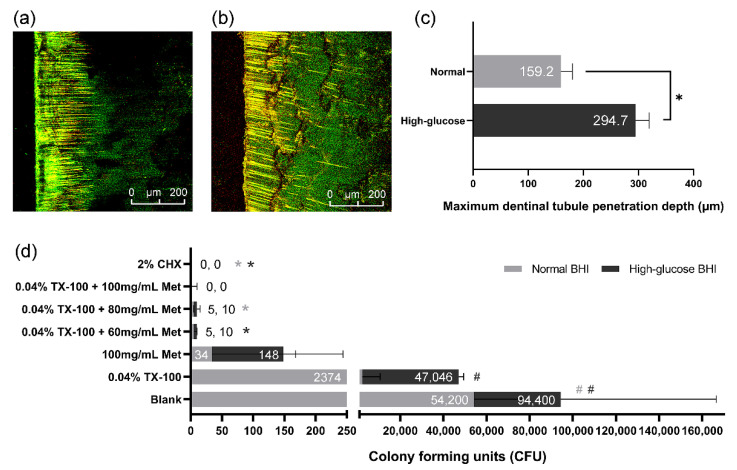
*E. faecalis* in dentinal tubules. (**a**,**b**) CLSM images (100×) of dentinal tubule invaded by *E. faecalis* in normal BHI and high-glucose BHI; (**c**) Mean tubule penetration depths of *E. faecalis*; (**d**) CFU counting of *E. faecalis* in dentin powder samples. (*: significant difference compared with the blank control group; #: significant difference compared with the 2% CHX group; *p* < 0.05).

In this study, the maximum tubule penetration depth of *E. faecalis* in normal and high-glucose BHI of the 28-day dentin slices were 159.2 ± 21.16 μm and 294.7 ± 24.42 μm, examined by CLSM (Figure 5a,b). The result indicated that *E. faecalis* in high-glucose BHI had a greater tubule penetration depth than those in normal BHI (*p* < 0.05; Figure 5c). The CFU counting result of the dentin powder samples showed that there were still considerable live bacteria in dentinal tubules after 7-day gel treatment of blank control or single-drug groups. However, the live bacteria in the combination drug groups were significantly fewer than the blank control group (*p* < 0.05; Figure 5d). There was no statistical difference between the combination drug groups and the 2% CHX group (Figure 5d), and only few or no live bacteria were detected in these groups. 

### 3.4. In Vitro Gene Expression Assay

**Figure 6:**
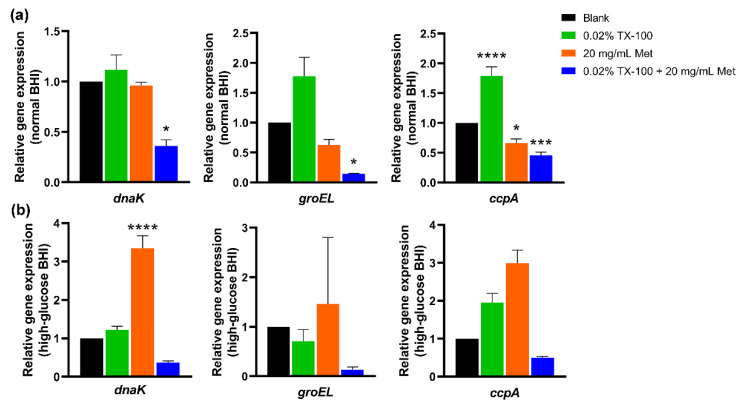
Relative gene expression of *dnaK*, *groEL*, and *ccpA* in *E. faecalis.* (**a**) in normal BHI; (**b**) in high-glucose BHI. (*: significant difference compared with the blank control group; * *p* < 0.05, *** *p* < 0.001, **** *p* < 0.0001).

The expression of stress response-related genes, *dnaK*, and *groEL* and carbon catabolite protein A (CcpA), were evaluated in this study. The results showed that the relative expression of *dnaK*, *groEL*, and *ccpA* in *E. faecalis* could be induced by TX-100 in normal BHI, and an upregulation of these genes was induced by Met in high-glucose BHI, compared to the blank control (*p* < 0.0001; Figure 6). However, when treated with the synergistic combination, the relative expression of the stress genes and *ccpA* was significantly suppressed in normal BHI (*p* < 0.05; Figure 6). 

### 3.5. Cytotoxicity Test

**Figure 7:**
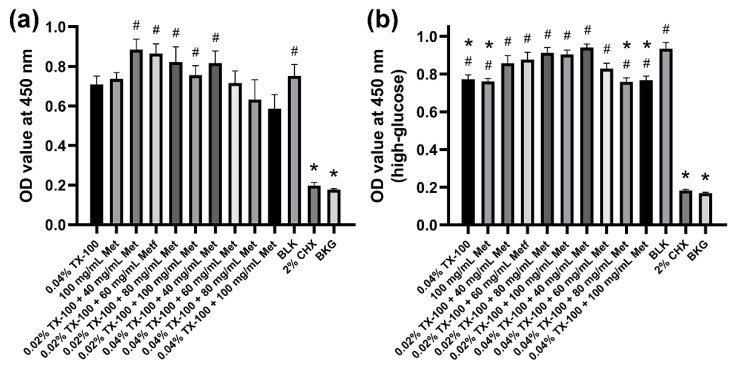
CCK-8 test on osteoblastic MC3T3-E1 cells. (**a**) in normal DMEM; (**b**) in high-glucose DMEM. (*: significant difference compared with the blank control group; #: significant difference compared with the 2% CHX group; *p* < 0.05).

Although cell activity slightly decreased with increasing drug concentrations, no significant difference was observed between most of the synergistic groups and the blank control. In addition, the cytotoxicity of the TX-100 + Met group was significantly lower than that of the 2% CHX group in both normal and high-glucose conditions (*p* < 0.05; Figure 7). 

## 4. Discussion

Previous studies have demonstrated a higher prevalence of periodontal and endodontic diseases [10], lower success rates of the root canal treatment [13], and slower periapical healing [12] in diabetic patients than in healthy controls. As a classical antihyperglycemic agent, Met, also shows pleiotropic effects as a clinical medicament. Studies have reported that Met can inhibit cellular inflammatory response by inhibiting the NF-κB signaling pathway [39], improve alveolar bone healing by increasing osteoblast differentiation in patients [40], and induce antibacterial effects against *P. gingivalis* in diabetes-related periodontitis [27]. However, there is still a lack of research on the root canal infection control in high-glucose condition, especially for *E. faecalis* infection. The results of this study, to our knowledge for the first time, confirmed that the antimicrobial activity of Met against *E. faecalis* could be greatly enhanced by combining it with a very low concentration of TX-100, in both normal and high-glucose conditions. 

For *E. faecalis* in high-glucose condition, previous studies have suggested that glucose can induce and enhance the expression of enterococcal surface protein [41], eDNA, and virulence-associated genes of *E. faecalis* [42], which are closely related to the enhanced biofilm matrix production and pathogenicity as well as a significantly higher drug resistance rate [43]. The above results suggest that a high-glucose concentration may enhance the pathogenicity and drug resistance of bacteria and ultimately lead to the failure of a clinical antibacterial treatment for diabetes. Thus, it is of great necessity to investigate bacterial infection control in high-glucose condition. 

When exposed to heat shock or other environmental stresses, the synthesis of a group of highly conserved heat shock proteins (HSPs or stress proteins) in cells rapidly increases [44]. In the general stress response of *E. faecalis*, HSP70/DnaK and HSP60/GroEL contribute to the refolding and degradation of denatured proteins to facilitate cell survival [45]. The transcription factor CcpA is a major regulator of genes expressed in different gram-positive bacteria. Leboeuf et al. [46] claimed that the inactivation of CcpA in *E. faecalis* leads to a slower growth rate and lower glycolytic capacity. Furthermore, Seidl et al. [47] pointed out that CcpA inactivation significantly reduced the drug resistance level of highly methicillin-resistant *S. aureus*. The RT-qPCR results of this study indicated that the expression of *danK, groEL, and ccpA* was significantly downregulated by the combination of TX-100 and Met in normal BHI. In this study, the MIC and MBC_99_ values of Met on *E. faecalis* were dramatically decreased in the presence of TX-100, displaying a higher susceptibility of *E. faecalis* to Met. In summary, we could surmise that by suppressing the expression of stress genes and CcpA, the combination of TX-100 and Met inhibited the stress response and glycolytic capacity of *E. faecalis*, thereby reduced their viability and proliferation and weakened their pathogenicity and drug resistance. Therefore, in the presence of TX-100, a much lower concentration of Met can still be significantly antimicrobial effective.

TX-100, usually used to solubilize proteins [48,49] or other macromolecules, can easily cross lipid bilayers and disrupt the lipid–lipid, protein–lipid, and protein–protein associations [50], thereby induce dynamic bilayer membrane perforation [51]. This mechanism increases cell membrane permeability and greatly facilitates the entry of more Met into the cell. Simultaneously, Met suppresses the glycolytic capacity and greatly blocks the energy source of bacteria by downregulating CcpA, ultimately disrupting normal physiological activity and accelerating the death process of *E. faecalis*. It may be another potential synergistic antimicrobial mechanism of the combination of TX-100 and Met. The FE-SEM images of the destroyed cell membrane of *E. faecalis* in the synergistic groups in this study confirmed the above statement.

It is worth noting that, besides the biofilm formation ability, *E. faecalis* also has a strong tubule invasion capacity. The depth of tubule invasion of *E. faecalis* in dentin biofilm model in the previous studies ranges from 156.2 ± 25.3 μm [52] to 1000 μm [53]. In this study, the results of CV assay confirmed the antibiofilm effect of the combination drug on immature biofilm, while the FE-SEM images displayed the abnormal morphology of *E. faecalis* in mature dentin biofilm after 7-day gel treatment. In addition, the OD value and CFU count also confirmed that no *E. faecalis* on the dentin surface survived the gel treatment. However, this effect may be limited by the poor permeability into the dentinal tubules, as the drug gel may not reach the dentinal tubules at the optimal effective concentration. As a result, live bacteria can still be detected from the dentinal tubules, which may cause root canal reinfection. This reminds us that a gel treatment could be antimicrobial effective on the bacteria on the dentin surface, but it may be limited effective for the bacteria that invade into the dentinal tubules, especially when there is a significantly greater penetration depth of *E. faecalis* in high-glucose condition.

Through the above-mentioned possible mechanisms, TX-100 improved the antibacterial activity of Met and displayed a synergistic antimicrobial effect against planktonic *E. faecalis* and in vitro dentin biofilm in both normal and high-glucose conditions. However, the in-depth molecular mechanism underlying the synergistic antimicrobial effect of the combinations of TX-100 and Met still needs to be further investigated. As mentioned above, for future in vivo applications, a drug-loaded biomaterial with controlled-release profile that can penetrate into the dentinal tubules may be a promising intracanal medication, which also calls for further investigations.

## 5. Conclusions

To our knowledge, this study is the first to combine TX-100 and Met to obtain both low cytotoxicity and enhanced synergistic antimicrobial activity against *E. faecalis* in both normal and high-glucose conditions, thus providing an effective new root canal disinfectant for diabetic patients.

## Figures and Tables

**Table 1:** Primer sequences used for the in vitro gene expression assay.

Gene		Primer Sequence	Amplicon Size (bp)	Reference
*16S*	*16Sf*	TCAAAGGAGAAGTTCGGGTCATTTCG	136	This study
	*16Sr*	TTCTTATGCTTGCGGTGGGACTTC		
*dnaK*	*dnaKf*	ACAGCCGGTGATAACAACCT	152	[38]
	*dnaKr*	TGGCAAGCTGATTTGTGTGC		
*groEL*	*groELf*	ACCTGATGAAACAGCAGCGA	136	[38]
	*groELr*	TGCTGGAGCCAACCCATTAG		
*ccpA*	*ccpAf*	AATAAGCGCATTGACACGGC	226	This study
	*ccpAr*	ATTTGGCTGATCGTGTCCGT		

**Table 2:** Minimum inhibitory concentration (MIC) and minimum bactericidal concentration (MBC_99_) values of Triton X-100 (TX-100) and metformin (Met) with or without TX-100 against *Enterococcus faecalis* (*E. faecalis*) in normal and high-glucose BHI.

Groups	*E. faecalis* in Normal BHI		*E. faecalis* in High-Glucose BHI	
	MIC	MBC_99_	MIC	MBC_99_
TX-100 (%*v*/*v*)	-	-	-	-
Met (mg/mL)	100	160	100	180
0.02% TX-100 + Met (mg/mL)	20	30	20	50
0.04% TX-100 + Met (mg/mL)	20	30	20	40

(-: No MIC or MBC_99_ could be detected.)

**Table 3:** The antibiofilm activities of TX-100 and Met with or without TX-100 against *E. faecalis* in normal and high-glucose BHI.

Groups	*E. faecalis* in Normal BHI		*E. faecalis* in High-Glucose BHI	
	MBIC_50_	MBRC_50_	MBIC_50_	MBRC_50_
TX-100 (%*v*/*v*)	-	-	-	-
Met (mg/mL)	100	200	100	300
0.02% TX-100 + Met (mg/mL)	40	40	40	60
0.04% TX-100 + Met (mg/mL)	10	40	20	60

(-: No MBIC_50_ or MBRC_50_ could be detected.)

## Data Availability

All data generated or analyzed during this study are included in this published article.
